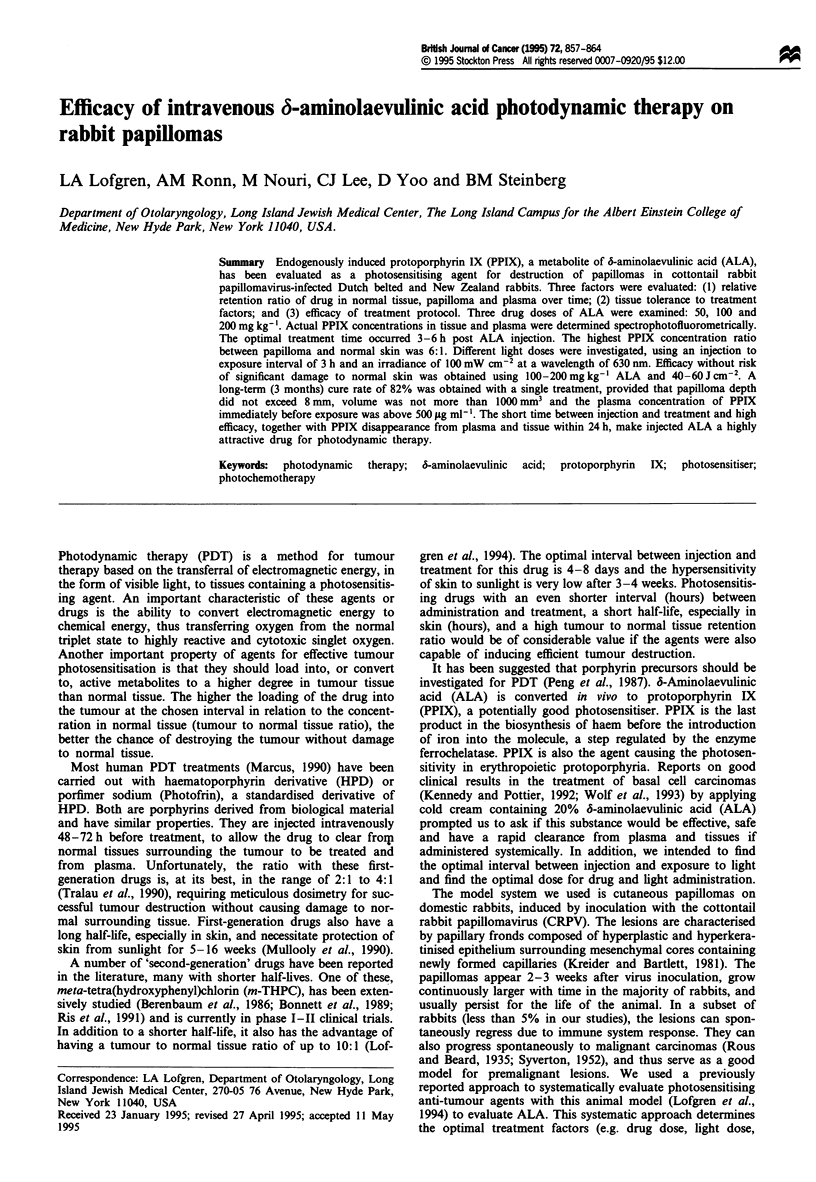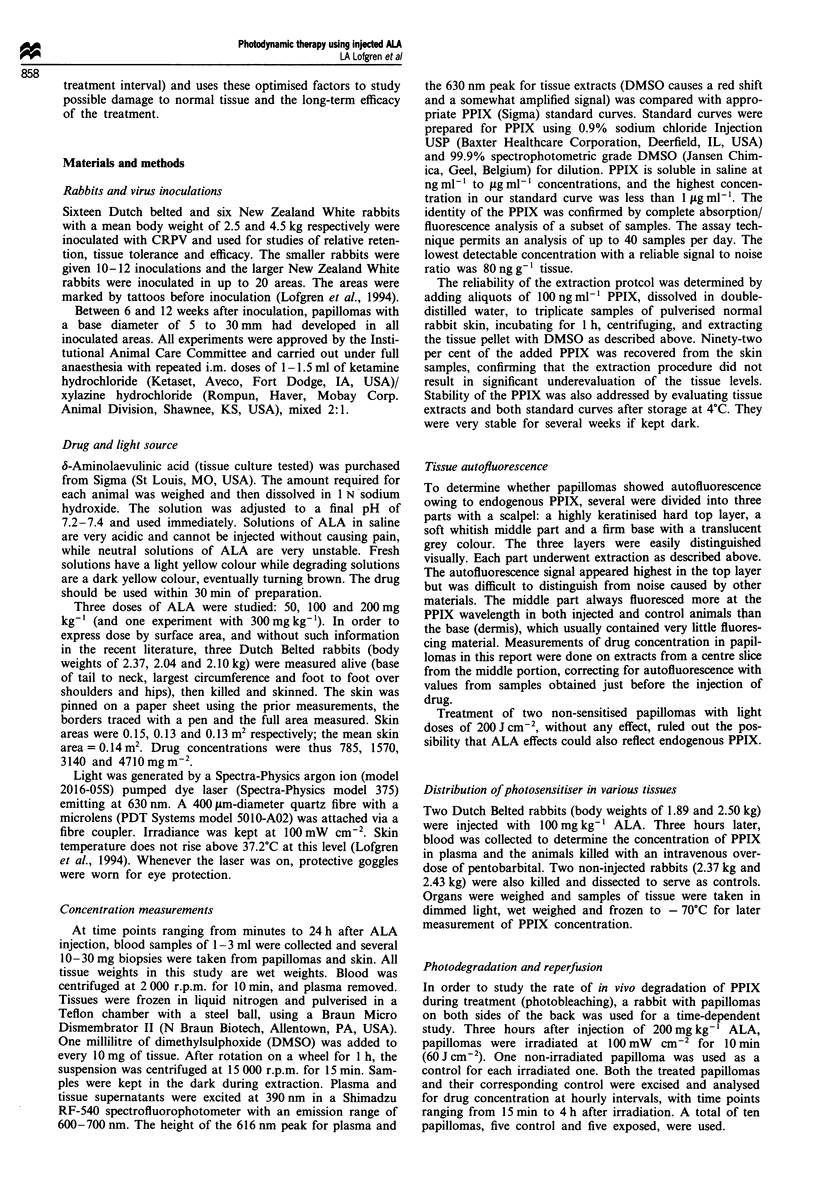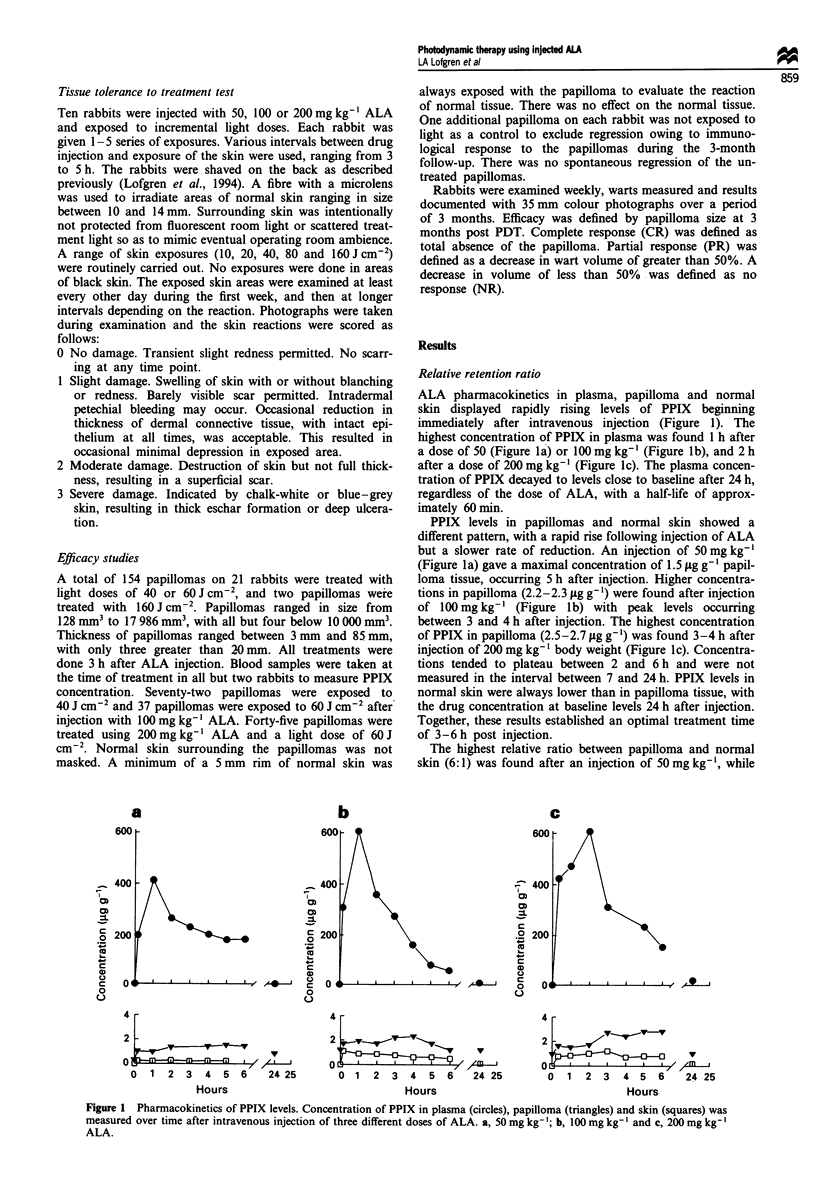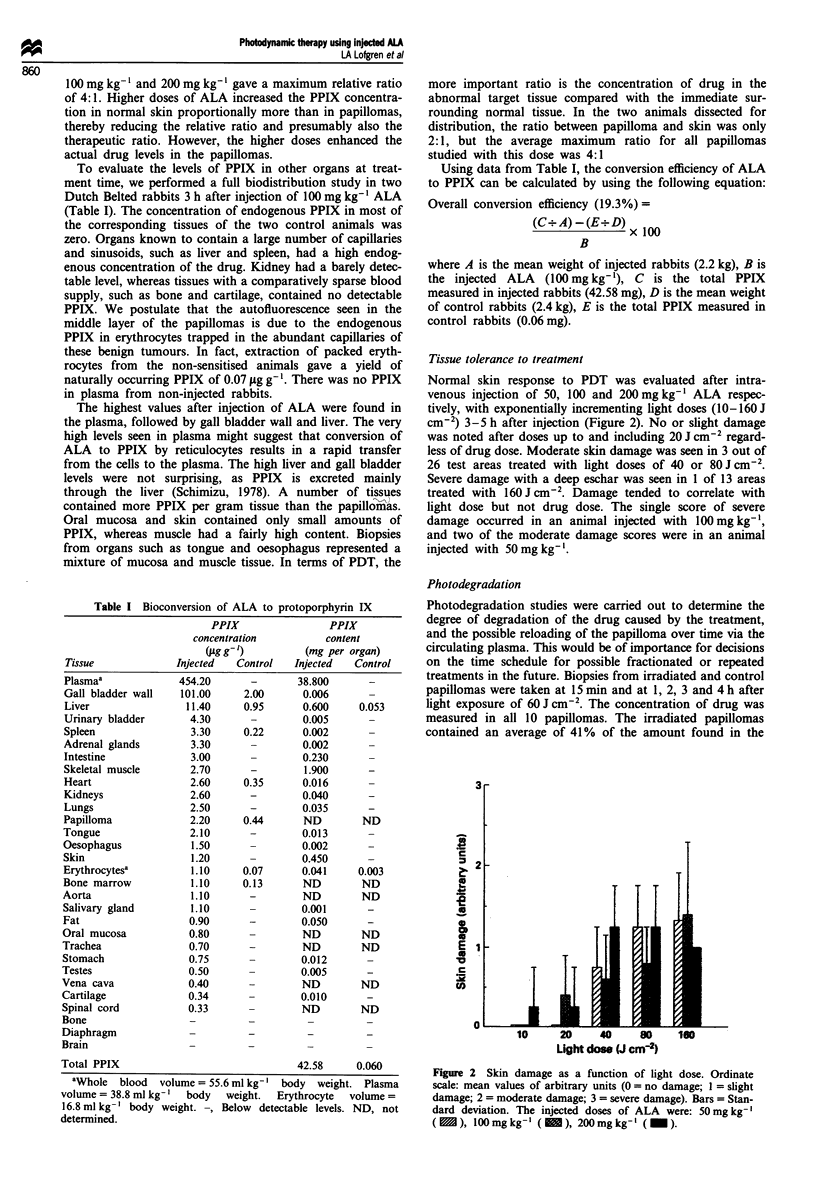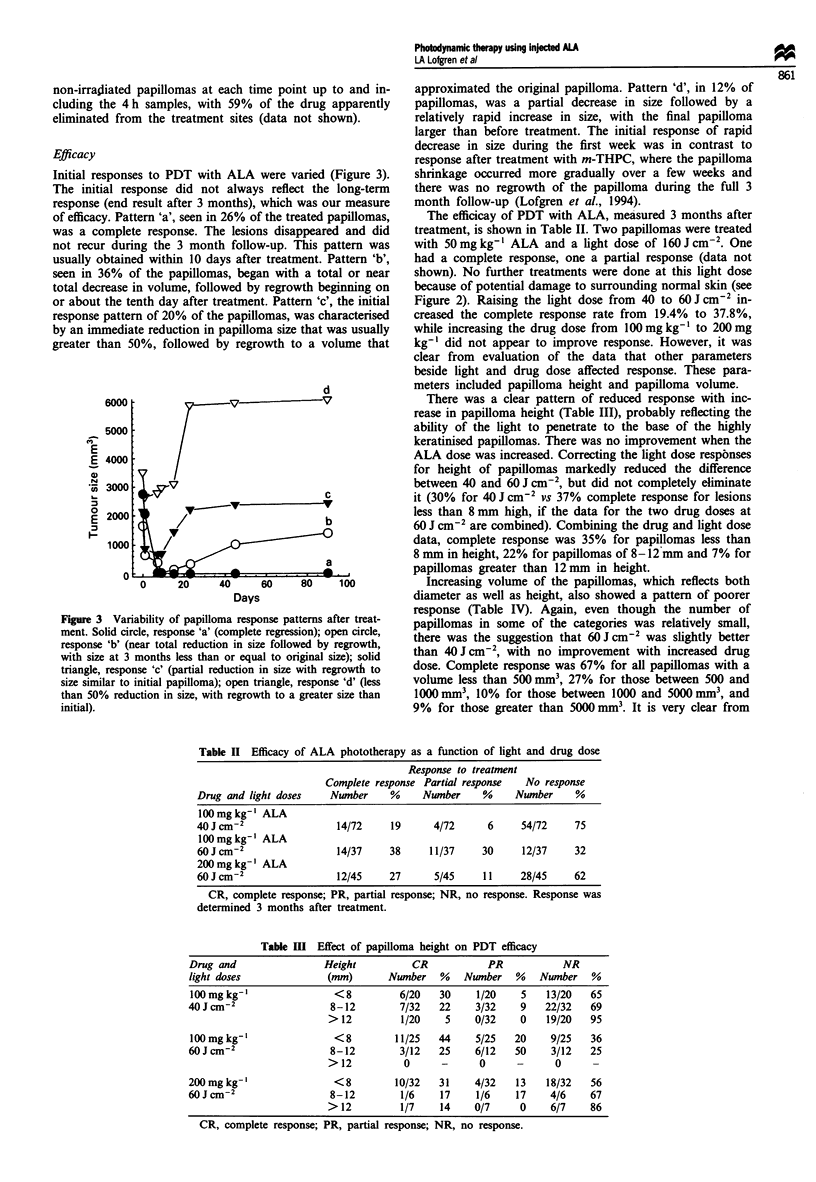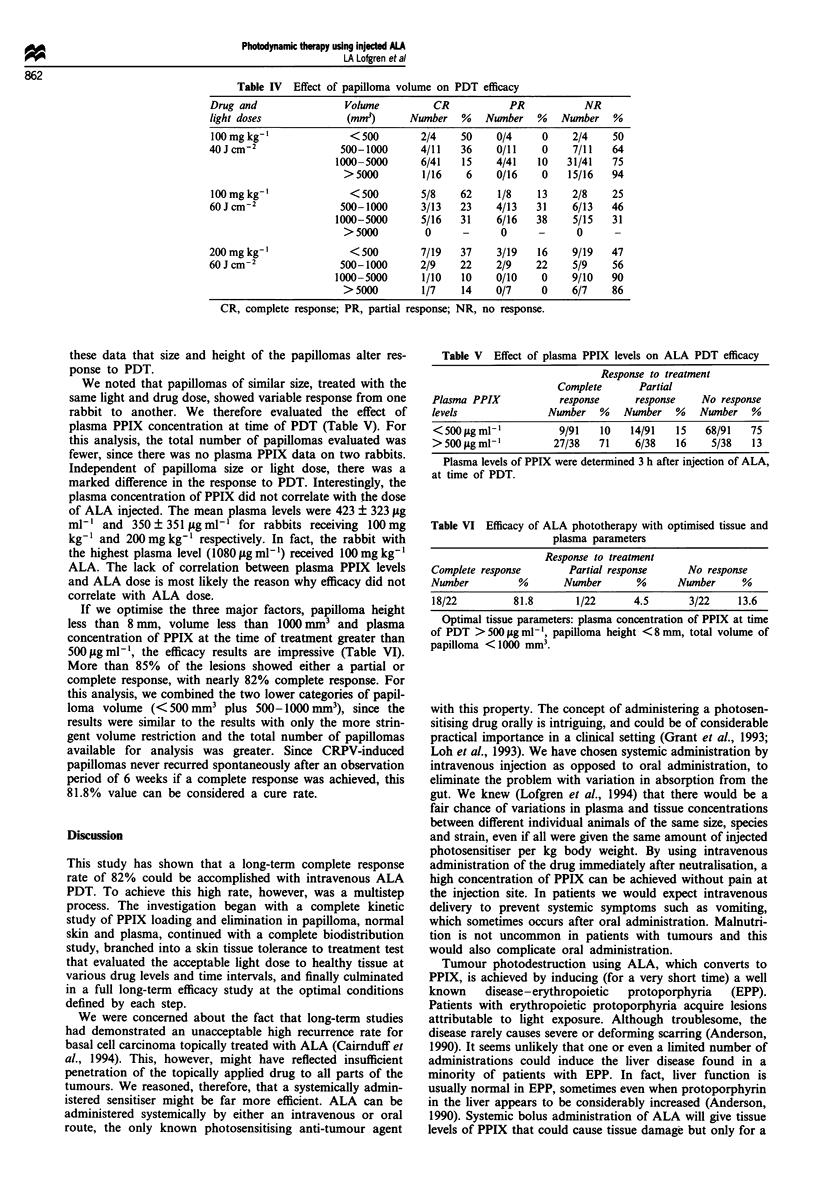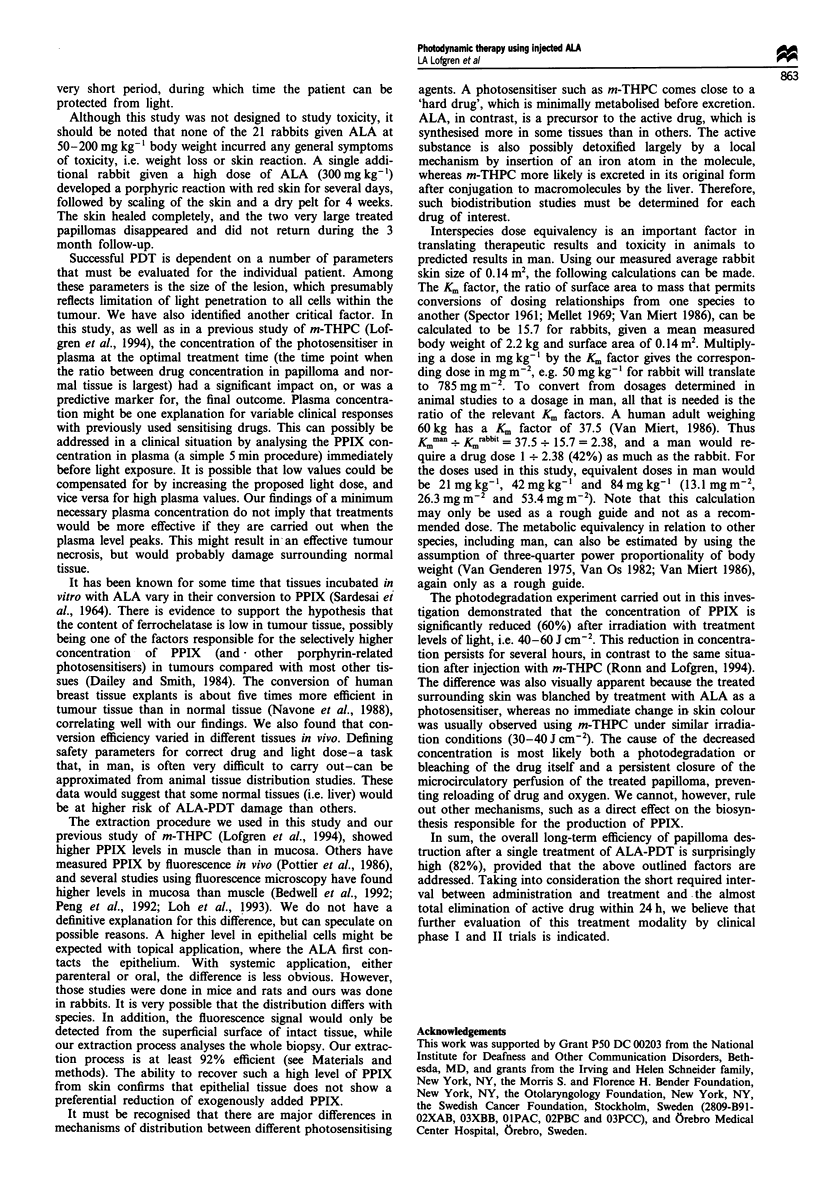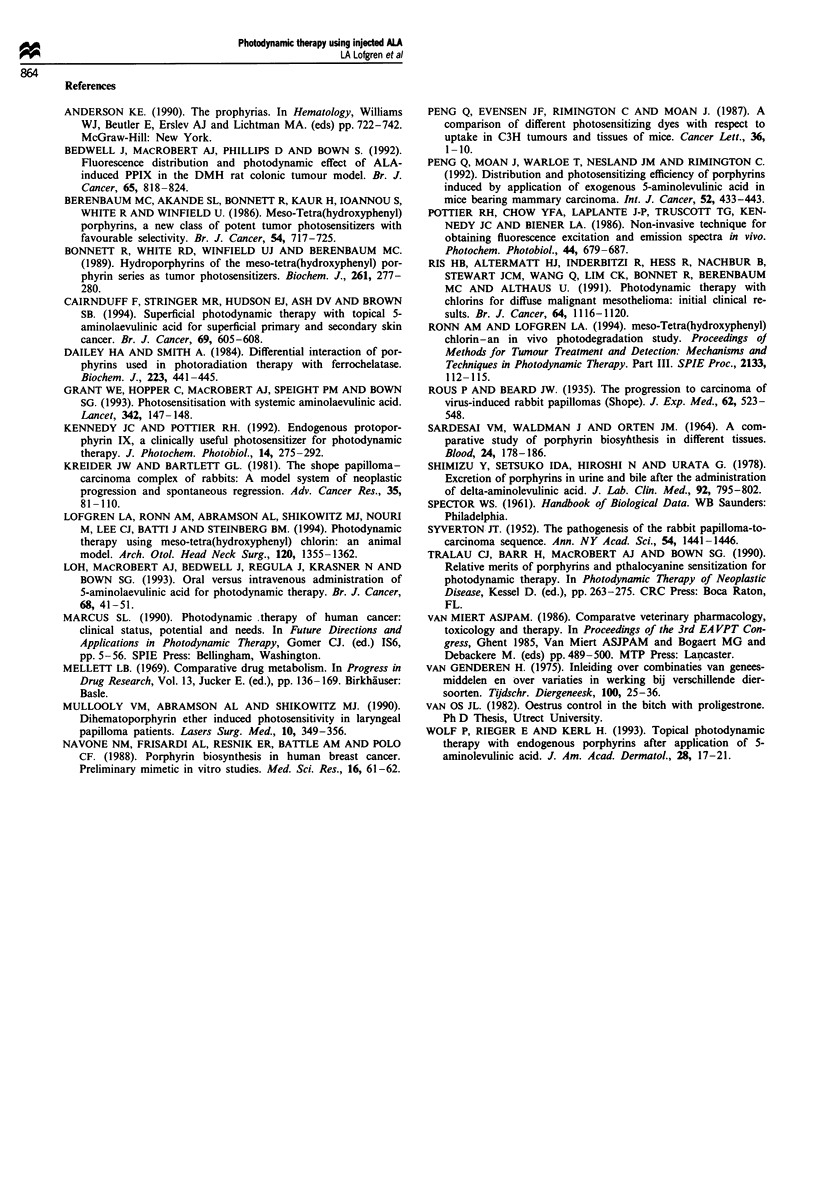# Efficacy of intravenous delta-aminolaevulinic acid photodynamic therapy on rabbit papillomas.

**DOI:** 10.1038/bjc.1995.424

**Published:** 1995-10

**Authors:** L. A. Lofgren, A. M. Ronn, M. Nouri, C. J. Lee, D. Yoo, B. M. Steinberg

**Affiliations:** Department of Otolaryngology, Long Island Jewish Medical Center, Albert Einstein College of Medicine, New Hyde Park, New York 11040, USA.

## Abstract

Endogenously induced protoporphyrin IX (PPIX), a metabolite of delta-aminolaevulinic acid (ALA), has been evaluated as a photosensitising agent for destruction of papillomas in cottontail rabbit papillomavirus-infected Dutch belted and New Zealand rabbits. Three factors were evaluated: (1) relative retention ratio of drug in normal tissue, papilloma and plasma over time; (2) tissue tolerance to treatment factors; and (3) efficacy of treatment protocol. Three drug doses of ALA were examined: 50, 100 and 200 mg kg-1. Actual PPIX concentrations in tissue and plasma were determined spectrophotofluorometrically. The optimal treatment time occurred 3-6 h post ALA injection. The highest PPIX concentration ratio between papilloma and normal skin was 6:1. Different light doses were investigated, using an injection to exposure interval of 3 h and an irradiance of 100 mW cm-2 at a wavelength of 630 nm. Efficacy without risk of significant damage to normal skin was obtained using 100-200 mg kg-1 ALA and 40-60 J cm-2. A long-term (3 months) cure rate of 82% was obtained with a single treatment, provided that papilloma depth did not exceed 8 mm, volume was not more than 1000 mm3 and the plasma concentration of PPIX immediately before exposure was above 500 micrograms ml-1. The short time between injection and treatment and high efficacy, together with PPIX disappearance from plasma and tissue within 24 h, make injected ALA a highly attractive drug for photodynamic therapy.


British Journal of Cancer (1995) 72, 857-864
© 1995 Stockton Press All rights reserved 0007-0920/95 $12.00

### Efficacy of intravenous δ-aminolaevulinic acid photodynamic therapy on rabbit papillomas

LA Lofgren, AM Ronn, M Nouri, CJ Lee, D Yoo and BM Steinberg

*Department of Otolaryngology, Long Island Jewish Medical Center, The Long Island Campus for the Albert Einstein College of Medicine, New Hyde Park, New York 11040, USA.*

**Summary** Endogenously induced protoporphyrin IX (PPIX), a metabolite of δ-aminolaevulinic acid (ALA), has been evaluated as a photosensitising agent for destruction of papillomas in cottontail rabbit papillomavirus-infected Dutch belted and New Zealand rabbits. Three factors were evaluated: (1) relative retention ratio of drug in normal tissue, papilloma and plasma over time; (2) tissue tolerance to treatment factors; and (3) efficacy of treatment protocol. Three drug doses of ALA were examined: 50, 100 and 200 mg $kg^{-1}$. Actual PPIX concentrations in tissue and plasma were determined spectrophotofluorometrically. The optimal treatment time occurred 3-6 h post ALA injection. The highest PPIX concentration ratio between papilloma and normal skin was 6:1. Different light doses were investigated, using an injection to exposure interval of 3 h and an irradiance of 100 mW $cm^{-2}$ at a wavelength of 630 nm. Efficacy without risk of significant damage to normal skin was obtained using 100-200 mg $kg^{-1}$ ALA and 40-60 J $cm^{-2}$. A long-term (3 months) cure rate of 82% was obtained with a single treatment, provided that papilloma depth did not exceed 8 mm, volume was not more than 1000 $mm^3$ and the plasma concentration of PPIX immediately before exposure was above 500 μg $ml^{-1}$. The short time between injection and treatment and high efficacy, together with PPIX disappearance from plasma and tissue within 24 h, make injected ALA a highly attractive drug for photodynamic therapy.

**Keywords:** photodynamic therapy; δ-aminolaevulinic acid; protoporphyrin IX; photosensitiser; photochemotherapy

Photodynamic therapy (PDT) is a method for tumour therapy based on the transferral of electromagnetic energy, in the form of visible light, to tissues containing a photosensitising agent. An important characteristic of these agents or drugs is the ability to convert electromagnetic energy to chemical energy, thus transferring oxygen from the normal triplet state to highly reactive and cytotoxic singlet oxygen. Another important property of agents for effective tumour photosensitisation is that they should load into, or convert to, active metabolites to a higher degree in tumour tissue than normal tissue. The higher the loading of the drug into the tumour at the chosen interval in relation to the concentration in normal tissue (tumour to normal tissue ratio), the better the chance of destroying the tumour without damage to normal tissue.

Most human PDT treatments (Marcus, 1990) have been carried out with haematoporphyrin derivative (HPD) or porfimer sodium (Photofrin), a standardised derivative of HPD. Both are porphyrins derived from biological material and have similar properties. They are injected intravenously 48-72 h before treatment, to allow the drug to clear from normal tissues surrounding the tumour to be treated and from plasma. Unfortunately, the ratio with these first-generation drugs is, at its best, in the range of 2:1 to 4:1 (Tralau *et al.*, 1990), requiring meticulous dosimetry for successful tumour destruction without causing damage to normal surrounding tissue. First-generation drugs also have a long half-life, especially in skin, and necessitate protection of skin from sunlight for 5-16 weeks (Mullooly *et al.*, 1990).

A number of 'second-generation' drugs have been reported in the literature, many with shorter half-lives. One of these, *meta*-tetra(hydroxyphenyl)chlorin (*m*-THPC), has been extensively studied (Berenbaum *et al.*, 1986; Bonnett *et al.*, 1989; Ris *et al.*, 1991) and is currently in phase I-II clinical trials. In addition to a shorter half-life, it also has the advantage of having a tumour to normal tissue ratio of up to 10:1 (Lofgren *et al.*, 1994). The optimal interval between injection and treatment for this drug is 4-8 days and the hypersensitivity of skin to sunlight is very low after 3-4 weeks. Photosensitising drugs with an even shorter interval (hours) between administration and treatment, a short half-life, especially in skin (hours), and a high tumour to normal tissue retention ratio would be of considerable value if the agents were also capable of inducing efficient tumour destruction.

It has been suggested that porphyrin precursors should be investigated for PDT (Peng *et al.*, 1987). δ-Aminolaevulinic acid (ALA) is converted *in vivo* to protoporphyrin IX (PPIX), a potentially good photosensitiser. PPIX is the last product in the biosynthesis of haem before the introduction of iron into the molecule, a step regulated by the enzyme ferrochelatase. PPIX is also the agent causing the photosensitivity in erythropoietic protoporphyria. Reports on good clinical results in the treatment of basal cell carcinomas (Kennedy and Pottier, 1992; Wolf *et al.*, 1993) by applying cold cream containing 20% δ-aminolaevulinic acid (ALA) prompted us to ask if this substance would be effective, safe and have a rapid clearance from plasma and tissues if administered systemically. In addition, we intended to find the optimal interval between injection and exposure to light and find the optimal dose for drug and light administration.

The model system we used is cutaneous papillomas on domestic rabbits, induced by inoculation with the cottontail rabbit papillomavirus (CRPV). The lesions are characterised by papillary fronds composed of hyperplastic and hyperkeratinised epithelium surrounding mesenchymal cores containing newly formed capillaries (Kreider and Bartlett, 1981). The papillomas appear 2-3 weeks after virus inoculation, grow continuously larger with time in the majority of rabbits, and usually persist for the life of the animal. In a subset of rabbits (less than 5% in our studies), the lesions can spontaneously regress due to immune system response. They can also progress spontaneously to malignant carcinomas (Rous and Beard, 1935; Syverton, 1952), and thus serve as a good model for premalignant lesions. We used a previously reported approach to systematically evaluate photosensitising anti-tumour agents with this animal model (Lofgren *et al.*, 1994) to evaluate ALA. This systematic approach determines the optimal treatment factors (e.g. drug dose, light dose,

Correspondence: LA Lofgren, Department of Otolaryngology, Long Island Jewish Medical Center, 270-05 76 Avenue, New Hyde Park, New York 11040, USA
Received 23 January 1995; revised 27 April 1995; accepted 11 May 1995

treatment interval) and uses these optimised factors to study possible damage to normal tissue and the long-term efficacy of the treatment.

### Materials and methods

### *Rabbits and virus inoculations*

Sixteen Dutch belted and six New Zealand White rabbits with a mean body weight of 2.5 and 4.5 kg respectively were inoculated with CRPV and used for studies of relative retention, tissue tolerance and efficacy. The smaller rabbits were given 10–12 inoculations and the larger New Zealand White rabbits were inoculated in up to 20 areas. The areas were marked by tattoos before inoculation (Lofgren *et al.*, 1994).

Between 6 and 12 weeks after inoculation, papillomas with a base diameter of 5 to 30 mm had developed in all inoculated areas. All experiments were approved by the Institutional Animal Care Committee and carried out under full anaesthesia with repeated i.m. doses of 1–1.5 ml of ketamine hydrochloride (Ketaset, Aveco, Fort Dodge, IA, USA)/ xylazine hydrochloride (Rompun, Haver, Mobay Corp. Animal Division, Shawnee, KS, USA), mixed 2:1.

### *Drug and light source*

δ-Aminolaevulinic acid (tissue culture tested) was purchased from Sigma (St Louis, MO, USA). The amount required for each animal was weighed and then dissolved in 1 N sodium hydroxide. The solution was adjusted to a final pH of 7.2–7.4 and used immediately. Solutions of ALA in saline are very acidic and cannot be injected without causing pain, while neutral solutions of ALA are very unstable. Fresh solutions have a light yellow colour while degrading solutions are a dark yellow colour, eventually turning brown. The drug should be used within 30 min of preparation.

Three doses of ALA were studied: 50, 100 and 200 mg $kg^{-1}$ (and one experiment with 300 mg $kg^{-1}$). In order to express dose by surface area, and without such information in the recent literature, three Dutch Belted rabbits (body weights of 2.37, 2.04 and 2.10 kg) were measured alive (base of tail to neck, largest circumference and foot to foot over shoulders and hips), then killed and skinned. The skin was pinned on a paper sheet using the prior measurements, the borders traced with a pen and the full area measured. Skin areas were 0.15, 0.13 and 0.13 $m^2$ respectively; the mean skin area = 0.14 $m^2$. Drug concentrations were thus 785, 1570, 3140 and 4710 mg $m^{-2}$.

Light was generated by a Spectra-Physics argon ion (model 2016-05S) pumped dye laser (Spectra-Physics model 375) emitting at 630 nm. A 400 μm-diameter quartz fibre with a microlens (PDT Systems model 5010-A02) was attached via a fibre coupler. Irradiance was kept at 100 mW $cm^{-2}$. Skin temperature does not rise above 37.2°C at this level (Lofgren *et al.*, 1994). Whenever the laser was on, protective goggles were worn for eye protection.

### *Concentration measurements*

At time points ranging from minutes to 24 h after ALA injection, blood samples of 1–3 ml were collected and several 10–30 mg biopsies were taken from papillomas and skin. All tissue weights in this study are wet weights. Blood was centrifuged at 2 000 r.p.m. for 10 min, and plasma removed. Tissues were frozen in liquid nitrogen and pulverised in a Teflon chamber with a steel ball, using a Braun Micro Dismembrator II (N Braun Biotech, Allentown, PA, USA). One millilitre of dimethylsulphoxide (DMSO) was added to every 10 mg of tissue. After rotation on a wheel for 1 h, the suspension was centrifuged at 15 000 r.p.m. for 15 min. Samples were kept in the dark during extraction. Plasma and tissue supernatants were excited at 390 nm in a Shimadzu RF-540 spectrofluorophotometer with an emission range of 600–700 nm. The height of the 616 nm peak for plasma and the 630 nm peak for tissue extracts (DMSO causes a red shift and a somewhat amplified signal) was compared with appropriate PPIX (Sigma) standard curves. Standard curves were prepared for PPIX using 0.9% sodium chloride Injection USP (Baxter Healthcare Corporation, Deerfield, IL, USA) and 99.9% spectrophotometric grade DMSO (Jansen Chimica, Geel, Belgium) for dilution. PPIX is soluble in saline at ng $ml^{-1}$ to μg $ml^{-1}$ concentrations, and the highest concentration in our standard curve was less than 1 μg $ml^{-1}$. The identity of the PPIX was confirmed by complete absorption/ fluorescence analysis of a subset of samples. The assay technique permits an analysis of up to 40 samples per day. The lowest detectable concentration with a reliable signal to noise ratio was 80 ng $g^{-1}$ tissue.

The reliability of the extraction protcol was determined by adding aliquots of 100 ng $ml^{-1}$ PPIX, dissolved in double-distilled water, to triplicate samples of pulverised normal rabbit skin, incubating for 1 h, centrifuging, and extracting the tissue pellet with DMSO as described above. Ninety-two per cent of the added PPIX was recovered from the skin samples, confirming that the extraction procedure did not result in significant underevaluation of the tissue levels. Stability of the PPIX was also addressed by evaluating tissue extracts and both standard curves after storage at 4°C. They were very stable for several weeks if kept dark.

### *Tissue autofluorescence*

To determine whether papillomas showed autofluorescence owing to endogenous PPIX, several were divided into three parts with a scalpel: a highly keratinised hard top layer, a soft whitish middle part and a firm base with a translucent grey colour. The three layers were easily distinguished visually. Each part underwent extraction as described above. The autofluorescence signal appeared highest in the top layer but was difficult to distinguish from noise caused by other materials. The middle part always fluoresced more at the PPIX wavelength in both injected and control animals than the base (dermis), which usually contained very little fluorescing material. Measurements of drug concentration in papillomas in this report were done on extracts from a centre slice from the middle portion, correcting for autofluorescence with values from samples obtained just before the injection of drug.

Treatment of two non-sensitised papillomas with light doses of 200 J $cm^{-2}$, without any effect, ruled out the possibility that ALA effects could also reflect endogenous PPIX.

### *Distribution of photosensitiser in various tissues*

Two Dutch Belted rabbits (body weights of 1.89 and 2.50 kg) were injected with 100 mg $kg^{-1}$ ALA. Three hours later, blood was collected to determine the concentration of PPIX in plasma and the animals killed with an intravenous overdose of pentobarbital. Two non-injected rabbits (2.37 kg and 2.43 kg) were also killed and dissected to serve as controls. Organs were weighed and samples of tissue were taken in dimmed light, wet weighed and frozen to – 70°C for later measurement of PPIX concentration.

### *Photodegradation and reperfusion*

In order to study the rate of *in vivo* degradation of PPIX during treatment (photobleaching), a rabbit with papillomas on both sides of the back was used for a time-dependent study. Three hours after injection of 200 mg $kg^{-1}$ ALA, papillomas were irradiated at 100 mW $cm^{-2}$ for 10 min (60 J $cm^{-2}$). One non-irradiated papilloma was used as a control for each irradiated one. Both the treated papillomas and their corresponding control were excised and analysed for drug concentration at hourly intervals, with time points ranging from 15 min to 4 h after irradiation. A total of ten papillomas, five control and five exposed, were used.

*Tissue tolerance to treatment test*

Ten rabbits were injected with 50, 100 or 200 mg $kg^{-1}$ ALA and exposed to incremental light doses. Each rabbit was given 1–5 series of exposures. Various intervals between drug injection and exposure of the skin were used, ranging from 3 to 5 h. The rabbits were shaved on the back as described previously (Lofgren *et al.*, 1994). A fibre with a microlens was used to irradiate areas of normal skin ranging in size between 10 and 14 mm. Surrounding skin was intentionally not protected from fluorescent room light or scattered treatment light so as to mimic eventual operating room ambience. A range of skin exposures (10, 20, 40, 80 and 160 J $cm^{-2}$) were routinely carried out. No exposures were done in areas of black skin. The exposed skin areas were examined at least every other day during the first week, and then at longer intervals depending on the reaction. Photographs were taken during examination and the skin reactions were scored as follows:

0 No damage. Transient slight redness permitted. No scarring at any time point.
1 Slight damage. Swelling of skin with or without blanching or redness. Barely visible scar permitted. Intradermal petechial bleeding may occur. Occasional reduction in thickness of dermal connective tissue, with intact epithelium at all times, was acceptable. This resulted in occasional minimal depression in exposed area.
2 Moderate damage. Destruction of skin but not full thickness, resulting in a superficial scar.
3 Severe damage. Indicated by chalk-white or blue-grey skin, resulting in thick eschar formation or deep ulceration.

*Efficacy studies*

A total of 154 papillomas on 21 rabbits were treated with light doses of 40 or 60 J $cm^{-2}$, and two papillomas were treated with 160 J $cm^{-2}$. Papillomas ranged in size from 128 $mm^3$ to 17 986 $mm^3$, with all but four below 10 000 $mm^3$. Thickness of papillomas ranged between 3 mm and 85 mm, with only three greater than 20 mm. All treatments were done 3 h after ALA injection. Blood samples were taken at the time of treatment in all but two rabbits to measure PPIX concentration. Seventy-two papillomas were exposed to 40 J $cm^{-2}$ and 37 papillomas were exposed to 60 J $cm^{-2}$ after injection with 100 mg $kg^{-1}$ ALA. Forty-five papillomas were treated using 200 mg $kg^{-1}$ ALA and a light dose of 60 J $cm^{-2}$. Normal skin surrounding the papillomas was not masked. A minimum of a 5 mm rim of normal skin was always exposed with the papilloma to evaluate the reaction of normal tissue. There was no effect on the normal tissue. One additional papilloma on each rabbit was not exposed to light as a control to exclude regression owing to immunological response to the papillomas during the 3-month follow-up. There was no spontaneous regression of the untreated papillomas.

Rabbits were examined weekly, warts measured and results documented with 35 mm colour photographs over a period of 3 months. Efficacy was defined by papilloma size at 3 months post PDT. Complete response (CR) was defined as total absence of the papilloma. Partial response (PR) was defined as a decrease in wart volume of greater than 50%. A decrease in volume of less than 50% was defined as no response (NR).

### Results

*Relative retention ratio*

ALA pharmacokinetics in plasma, papilloma and normal skin displayed rapidly rising levels of PPIX beginning immediately after intravenous injection (Figure 1). The highest concentration of PPIX in plasma was found 1 h after a dose of 50 (Figure 1a) or 100 mg $kg^{-1}$ (Figure 1b), and 2 h after a dose of 200 mg $kg^{-1}$ (Figure 1c). The plasma concentration of PPIX decayed to levels close to baseline after 24 h, regardless of the dose of ALA, with a half-life of approximately 60 min.

PPIX levels in papillomas and normal skin showed a different pattern, with a rapid rise following injection of ALA but a slower rate of reduction. An injection of 50 mg $kg^{-1}$ (Figure 1a) gave a maximal concentration of 1.5 $\mu g\ g^{-1}$ papilloma tissue, occurring 5 h after injection. Higher concentrations in papilloma (2.2–2.3 $\mu g\ g^{-1}$) were found after injection of 100 mg $kg^{-1}$ (Figure 1b) with peak levels occurring between 3 and 4 h after injection. The highest concentration of PPIX in papilloma (2.5–2.7 $\mu g\ g^{-1}$) was found 3–4 h after injection of 200 mg $kg^{-1}$ body weight (Figure 1c). Concentrations tended to plateau between 2 and 6 h and were not measured in the interval between 7 and 24 h. PPIX levels in normal skin were always lower than in papilloma tissue, with the drug concentration at baseline levels 24 h after injection. Together, these results established an optimal treatment time of 3–6 h post injection.

The highest relative ratio between papilloma and normal skin (6:1) was found after an injection of 50 mg $kg^{-1}$, while

**Figure 1** Pharmacokinetics of PPIX levels. Concentration of PPIX in plasma (circles), papilloma (triangles) and skin (squares) was measured over time after intravenous injection of three different doses of ALA. **a**, 50 mg $kg^{-1}$; **b**, 100 mg $kg^{-1}$ and **c**, 200 mg $kg^{-1}$ ALA.

100 mg kg$^{-1}$ and 200 mg kg$^{-1}$ gave a maximum relative ratio of 4:1. Higher doses of ALA increased the PPIX concentration in normal skin proportionally more than in papillomas, thereby reducing the relative ratio and presumably also the therapeutic ratio. However, the higher doses enhanced the actual drug levels in the papillomas.

To evaluate the levels of PPIX in other organs at treatment time, we performed a full biodistribution study in two Dutch Belted rabbits 3 h after injection of 100 mg kg$^{-1}$ ALA (Table I). The concentration of endogenous PPIX in most of the corresponding tissues of the two control animals was zero. Organs known to contain a large number of capillaries and sinusoids, such as liver and spleen, had a high endogenous concentration of the drug. Kidney had a barely detectable level, whereas tissues with a comparatively sparse blood supply, such as bone and cartilage, contained no detectable PPIX. We postulate that the autofluorescence seen in the middle layer of the papillomas is due to the endogenous PPIX in erythrocytes trapped in the abundant capillaries of these benign tumours. In fact, extraction of packed erythrocytes from the non-sensitised animals gave a yield of naturally occurring PPIX of 0.07 µg g$^{-1}$. There was no PPIX in plasma from non-injected rabbits.

The highest values after injection of ALA were found in the plasma, followed by gall bladder wall and liver. The very high levels seen in plasma might suggest that conversion of ALA to PPIX by reticulocytes results in a rapid transfer from the cells to the plasma. The high liver and gall bladder levels were not surprising, as PPIX is excreted mainly through the liver (Schimizu, 1978). A number of tissues contained more PPIX per gram tissue than the papillomas. Oral mucosa and skin contained only small amounts of PPIX, whereas muscle had a fairly high content. Biopsies from organs such as tongue and oesophagus represented a mixture of mucosa and muscle tissue. In terms of PDT, the more important ratio is the concentration of drug in the abnormal target tissue compared with the immediate surrounding normal tissue. In the two animals dissected for distribution, the ratio between papilloma and skin was only 2:1, but the average maximum ratio for all papillomas studied with this dose was 4:1

Using data from Table I, the conversion efficiency of ALA to PPIX can be calculated by using the following equation:

Overall conversion efficiency (19.3%) =

$$\frac{(C \div A) - (E \div D)}{B} \times 100$$

where $A$ is the mean weight of injected rabbits (2.2 kg), $B$ is the injected ALA (100 mg kg$^{-1}$), $C$ is the total PPIX measured in injected rabbits (42.58 mg), $D$ is the mean weight of control rabbits (2.4 kg), $E$ is the total PPIX measured in control rabbits (0.06 mg).

**Table I** Bioconversion of ALA to protoporphyrin IX

| *Tissue* | *PPIX concentration (µg g$^{-1}$)* Injected | Control | *PPIX content (mg per organ)* Injected | Control |
|---|---|---|---|---|
| Plasma[a] | 454.20 | – | 38.800 | – |
| Gall bladder wall | 101.00 | 2.00 | 0.006 | – |
| Liver | 11.40 | 0.95 | 0.600 | 0.053 |
| Urinary bladder | 4.30 | – | 0.005 | – |
| Spleen | 3.30 | 0.22 | 0.002 | – |
| Adrenal glands | 3.30 | – | 0.002 | – |
| Intestine | 3.00 | – | 0.230 | – |
| Skeletal muscle | 2.70 | – | 1.900 | – |
| Heart | 2.60 | 0.35 | 0.016 | – |
| Kidneys | 2.60 | – | 0.040 | – |
| Lungs | 2.50 | – | 0.035 | – |
| Papilloma | 2.20 | 0.44 | ND | ND |
| Tongue | 2.10 | – | 0.013 | – |
| Oesophagus | 1.50 | – | 0.002 | – |
| Skin | 1.20 | – | 0.450 | – |
| Erythrocytes[a] | 1.10 | 0.07 | 0.041 | 0.003 |
| Bone marrow | 1.10 | 0.13 | ND | ND |
| Aorta | 1.10 | – | ND | ND |
| Salivary gland | 1.10 | – | 0.001 | – |
| Fat | 0.90 | – | 0.050 | – |
| Oral mucosa | 0.80 | – | ND | ND |
| Trachea | 0.70 | – | ND | ND |
| Stomach | 0.75 | – | 0.012 | – |
| Testes | 0.50 | – | 0.005 | – |
| Vena cava | 0.40 | – | ND | ND |
| Cartilage | 0.34 | – | 0.010 | – |
| Spinal cord | 0.33 | – | ND | ND |
| Bone | – | – | – | – |
| Diaphragm | – | – | – | – |
| Brain | – | – | – | – |
| Total PPIX | | | 42.58 | 0.060 |

[a]Whole blood volume = 55.6 ml kg$^{-1}$ body weight. Plasma volume = 38.8 ml kg$^{-1}$ body weight. Erythrocyte volume = 16.8 ml kg$^{-1}$ body weight. –, Below detectable levels. ND, not determined.

*Tissue tolerance to treatment*

Normal skin response to PDT was evaluated after intravenous injection of 50, 100 and 200 mg kg$^{-1}$ ALA respectively, with exponentially incrementing light doses (10–160 J cm$^{-2}$) 3–5 h after injection (Figure 2). No or slight damage was noted after doses up to and including 20 J cm$^{-2}$ regardless of drug dose. Moderate skin damage was seen in 3 out of 26 test areas treated with light doses of 40 or 80 J cm$^{-2}$. Severe damage with a deep eschar was seen in 1 of 13 areas treated with 160 J cm$^{-2}$. Damage tended to correlate with light dose but not drug dose. The single score of severe damage occurred in an animal injected with 100 mg kg$^{-1}$, and two of the moderate damage scores were in an animal injected with 50 mg kg$^{-1}$.

*Photodegradation*

Photodegradation studies were carried out to determine the degree of degradation of the drug caused by the treatment, and the possible reloading of the papilloma over time via the circulating plasma. This would be of importance for decisions on the time schedule for possible fractionated or repeated treatments in the future. Biopsies from irradiated and control papillomas were taken at 15 min and at 1, 2, 3 and 4 h after light exposure of 60 J cm$^{-2}$. The concentration of drug was measured in all 10 papillomas. The irradiated papillomas contained an average of 41% of the amount found in the

**Figure 2** Skin damage as a function of light dose. Ordinate scale: mean values of arbitrary units (0 = no damage; 1 = slight damage; 2 = moderate damage; 3 = severe damage). Bars = Standard deviation. The injected doses of ALA were: 50 mg kg$^{-1}$ (▨), 100 mg kg$^{-1}$ (▩), 200 mg kg$^{-1}$ (■).

non-irradiated papillomas at each time point up to and including the 4 h samples, with 59% of the drug apparently eliminated from the treatment sites (data not shown).

### *Efficacy*

Initial responses to PDT with ALA were varied (Figure 3). The initial response did not always reflect the long-term response (end result after 3 months), which was our measure of efficacy. Pattern 'a', seen in 26% of the treated papillomas, was a complete response. The lesions disappeared and did not recur during the 3 month follow-up. This pattern was usually obtained within 10 days after treatment. Pattern 'b', seen in 36% of the papillomas, began with a total or near total decrease in volume, followed by regrowth beginning on or about the tenth day after treatment. Pattern 'c', the initial response pattern of 20% of the papillomas, was characterised by an immediate reduction in papilloma size that was usually greater than 50%, followed by regrowth to a volume that approximated the original papilloma. Pattern 'd', in 12% of papillomas, was a partial decrease in size followed by a relatively rapid increase in size, with the final papilloma larger than before treatment. The initial response of rapid decrease in size during the first week was in contrast to response after treatment with *m*-THPC, where the papilloma shrinkage occurred more gradually over a few weeks and there was no regrowth of the papilloma during the full 3 month follow-up (Lofgren *et al.*, 1994).

**Figure 3** Variability of papilloma response patterns after treatment. Solid circle, response 'a' (complete regression); open circle, response 'b' (near total reduction in size followed by regrowth, with size at 3 months less than or equal to original size); solid triangle, response 'c' (partial reduction in size with regrowth to size similar to initial papilloma); open triangle, response 'd' (less than 50% reduction in size, with regrowth to a greater size than initial).

The efficicay of PDT with ALA, measured 3 months after treatment, is shown in Table II. Two papillomas were treated with 50 mg kg$^{-1}$ ALA and a light dose of 160 J cm$^{-2}$. One had a complete response, one a partial response (data not shown). No further treatments were done at this light dose because of potential damage to surrounding normal skin (see Figure 2). Raising the light dose from 40 to 60 J cm$^{-2}$ increased the complete response rate from 19.4% to 37.8%, while increasing the drug dose from 100 mg kg$^{-1}$ to 200 mg kg$^{-1}$ did not appear to improve response. However, it was clear from evaluation of the data that other parameters beside light and drug dose affected response. These parameters included papilloma height and papilloma volume.

There was a clear pattern of reduced response with increase in papilloma height (Table III), probably reflecting the ability of the light to penetrate to the base of the highly keratinised papillomas. There was no improvement when the ALA dose was increased. Correcting the light dose responses for height of papillomas markedly reduced the difference between 40 and 60 J cm$^{-2}$, but did not completely eliminate it (30% for 40 J cm$^{-2}$ *vs* 37% complete response for lesions less than 8 mm high, if the data for the two drug doses at 60 J cm$^{-2}$ are combined). Combining the drug and light dose data, complete response was 35% for papillomas less than 8 mm in height, 22% for papillomas of 8–12 mm and 7% for papillomas greater than 12 mm in height.

Increasing volume of the papillomas, which reflects both diameter as well as height, also showed a pattern of poorer response (Table IV). Again, even though the number of papillomas in some of the categories was relatively small, there was the suggestion that 60 J cm$^{-2}$ was slightly better than 40 J cm$^{-2}$, with no improvement with increased drug dose. Complete response was 67% for all papillomas with a volume less than 500 mm$^3$, 27% for those between 500 and 1000 mm$^3$, 10% for those between 1000 and 5000 mm$^3$, and 9% for those greater than 5000 mm$^3$. It is very clear from

**Table II** Efficacy of ALA phototherapy as a function of light and drug dose

| | *Response to treatment* | | | | | |
|---|---|---|---|---|---|---|
| | *Complete response* | | *Partial response* | | *No response* | |
| *Drug and light doses* | *Number* | *%* | *Number* | *%* | *Number* | *%* |
| 100 mg kg$^{-1}$ ALA 40 J cm$^{-2}$ | 14/72 | 19 | 4/72 | 6 | 54/72 | 75 |
| 100 mg kg$^{-1}$ ALA 60 J cm$^{-2}$ | 14/37 | 38 | 11/37 | 30 | 12/37 | 32 |
| 200 mg kg$^{-1}$ ALA 60 J cm$^{-2}$ | 12/45 | 27 | 5/45 | 11 | 28/45 | 62 |

CR, complete response; PR, partial response; NR, no response. Response was determined 3 months after treatment.

**Table III** Effect of papilloma height on PDT efficacy

| *Drug and light doses* | *Height (mm)* | *CR Number* | *%* | *PR Number* | *%* | *NR Number* | *%* |
|---|---|---|---|---|---|---|---|
| 100 mg kg$^{-1}$ 40 J cm$^{-2}$ | <8 | 6/20 | 30 | 1/20 | 5 | 13/20 | 65 |
| | 8–12 | 7/32 | 22 | 3/32 | 9 | 22/32 | 69 |
| | >12 | 1/20 | 5 | 0/32 | 0 | 19/20 | 95 |
| 100 mg kg$^{-1}$ 60 J cm$^{-2}$ | <8 | 11/25 | 44 | 5/25 | 20 | 9/25 | 36 |
| | 8–12 | 3/12 | 25 | 6/12 | 50 | 3/12 | 25 |
| | >12 | 0 | – | 0 | – | 0 | – |
| 200 mg kg$^{-1}$ 60 J cm$^{-2}$ | <8 | 10/32 | 31 | 4/32 | 13 | 18/32 | 56 |
| | 8–12 | 1/6 | 17 | 1/6 | 17 | 4/6 | 67 |
| | >12 | 1/7 | 14 | 0/7 | 0 | 6/7 | 86 |

CR, complete response; PR, partial response; NR, no response.

Table IV Effect of papilloma volume on PDT efficacy

| *Drug and light doses* | *Volume (mm³)* | *CR Number* | *%* | *PR Number* | *%* | *NR Number* | *%* |
|---|---|---|---|---|---|---|---|
| 100 mg kg⁻¹ | <500 | 2/4 | 50 | 0/4 | 0 | 2/4 | 50 |
| 40 J cm⁻² | 500–1000 | 4/11 | 36 | 0/11 | 0 | 7/11 | 64 |
| | 1000–5000 | 6/41 | 15 | 4/41 | 10 | 31/41 | 75 |
| | >5000 | 1/16 | 6 | 0/16 | 0 | 15/16 | 94 |
| 100 mg kg⁻¹ | <500 | 5/8 | 62 | 1/8 | 13 | 2/8 | 25 |
| 60 J cm⁻² | 500–1000 | 3/13 | 23 | 4/13 | 31 | 6/13 | 46 |
| | 1000–5000 | 5/16 | 31 | 6/16 | 38 | 5/15 | 31 |
| | >5000 | 0 | – | 0 | – | 0 | – |
| 200 mg kg⁻¹ | <500 | 7/19 | 37 | 3/19 | 16 | 9/19 | 47 |
| 60 J cm⁻² | 500–1000 | 2/9 | 22 | 2/9 | 22 | 5/9 | 56 |
| | 1000–5000 | 1/10 | 10 | 0/10 | 0 | 9/10 | 90 |
| | >5000 | 1/7 | 14 | 0/7 | 0 | 6/7 | 86 |

CR, complete response; PR, partial response; NR, no response.

these data that size and height of the papillomas alter response to PDT.

We noted that papillomas of similar size, treated with the same light and drug dose, showed variable response from one rabbit to another. We therefore evaluated the effect of plasma PPIX concentration at time of PDT (Table V). For this analysis, the total number of papillomas evaluated was fewer, since there was no plasma PPIX data on two rabbits. Independent of papilloma size or light dose, there was a marked difference in the response to PDT. Interestingly, the plasma concentration of PPIX did not correlate with the dose of ALA injected. The mean plasma levels were 423 ± 323 μg ml⁻¹ and 350 ± 351 μg ml⁻¹ for rabbits receiving 100 mg kg⁻¹ and 200 mg kg⁻¹ respectively. In fact, the rabbit with the highest plasma level (1080 μg ml⁻¹) received 100 mg kg⁻¹ ALA. The lack of correlation between plasma PPIX levels and ALA dose is most likely the reason why efficacy did not correlate with ALA dose.

If we optimise the three major factors, papilloma height less than 8 mm, volume less than 1000 mm³ and plasma concentration of PPIX at the time of treatment greater than 500 μg ml⁻¹, the efficacy results are impressive (Table VI). More than 85% of the lesions showed either a partial or complete response, with nearly 82% complete response. For this analysis, we combined the two lower categories of papilloma volume (<500 mm³ plus 500–1000 mm³), since the results were similar to the results with only the more stringent volume restriction and the total number of papillomas available for analysis was greater. Since CRPV-induced papillomas never recurred spontaneously after an observation period of 6 weeks if a complete response was achieved, this 81.8% value can be considered a cure rate.

Table V Effect of plasma PPIX levels on ALA PDT efficacy

| *Plasma PPIX levels* | *Response to treatment: Complete response Number* | *%* | *Partial response Number* | *%* | *No response Number* | *%* |
|---|---|---|---|---|---|---|
| <500 μg ml⁻¹ | 9/91 | 10 | 14/91 | 15 | 68/91 | 75 |
| >500 μg ml⁻¹ | 27/38 | 71 | 6/38 | 16 | 5/38 | 13 |

Plasma levels of PPIX were determined 3 h after injection of ALA, at time of PDT.

Table VI Efficacy of ALA phototherapy with optimised tissue and plasma parameters

| *Response to treatment: Complete response Number* | *%* | *Partial response Number* | *%* | *No response Number* | *%* |
|---|---|---|---|---|---|
| 18/22 | 81.8 | 1/22 | 4.5 | 3/22 | 13.6 |

Optimal tissue parameters: plasma concentration of PPIX at time of PDT >500 μg ml⁻¹, papilloma height <8 mm, total volume of papilloma <1000 mm³.

### Discussion

This study has shown that a long-term complete response rate of 82% could be accomplished with intravenous ALA PDT. To achieve this high rate, however, was a multistep process. The investigation began with a complete kinetic study of PPIX loading and elimination in papilloma, normal skin and plasma, continued with a complete biodistribution study, branched into a skin tissue tolerance to treatment test that evaluated the acceptable light dose to healthy tissue at various drug levels and time intervals, and finally culminated in a full long-term efficacy study at the optimal conditions defined by each step.

We were concerned about the fact that long-term studies had demonstrated an unacceptable high recurrence rate for basal cell carcinoma topically treated with ALA (Cairnduff *et al.*, 1994). This, however, might have reflected insufficient penetration of the topically applied drug to all parts of the tumours. We reasoned, therefore, that a systemically administered sensitiser might be far more efficient. ALA can be administered systemically by either an intravenous or oral route, the only known photosensitising anti-tumour agent with this property. The concept of administering a photosensitising drug orally is intriguing, and could be of considerable practical importance in a clinical setting (Grant *et al.*, 1993; Loh *et al.*, 1993). We have chosen systemic administration by intravenous injection as opposed to oral administration, to eliminate the problem with variation in absorption from the gut. We knew (Lofgren *et al.*, 1994) that there would be a fair chance of variations in plasma and tissue concentrations between different individual animals of the same size, species and strain, even if all were given the same amount of injected photosensitiser per kg body weight. By using intravenous administration of the drug immediately after neutralisation, a high concentration of PPIX can be achieved without pain at the injection site. In patients we would expect intravenous delivery to prevent systemic symptoms such as vomiting, which sometimes occurs after oral administration. Malnutrition is not uncommon in patients with tumours and this would also complicate oral administration.

Tumour photodestruction using ALA, which converts to PPIX, is achieved by inducing (for a very short time) a well known disease–erythropoietic protoporphyria (EPP). Patients with erythropoietic protoporphyria acquire lesions attributable to light exposure. Although troublesome, the disease rarely causes severe or deforming scarring (Anderson, 1990). It seems unlikely that one or even a limited number of administrations could induce the liver disease found in a minority of patients with EPP. In fact, liver function is usually normal in EPP, sometimes even when protoporphyrin in the liver appears to be considerably increased (Anderson, 1990). Systemic bolus administration of ALA will give tissue levels of PPIX that could cause tissue damage but only for a very short period, during which time the patient can be protected from light.

Although this study was not designed to study toxicity, it should be noted that none of the 21 rabbits given ALA at 50–200 mg kg$^{-1}$ body weight incurred any general symptoms of toxicity, i.e. weight loss or skin reaction. A single additional rabbit given a high dose of ALA (300 mg kg$^{-1}$) developed a porphyric reaction with red skin for several days, followed by scaling of the skin and a dry pelt for 4 weeks. The skin healed completely, and the two very large treated papillomas disappeared and did not return during the 3 month follow-up.

Successful PDT is dependent on a number of parameters that must be evaluated for the individual patient. Among these parameters is the size of the lesion, which presumably reflects limitation of light penetration to all cells within the tumour. We have also identified another critical factor. In this study, as well as in a previous study of *m*-THPC (Lofgren *et al.*, 1994), the concentration of the photosensitiser in plasma at the optimal treatment time (the time point when the ratio between drug concentration in papilloma and normal tissue is largest) had a significant impact on, or was a predictive marker for, the final outcome. Plasma concentration might be one explanation for variable clinical responses with previously used sensitising drugs. This can possibly be addressed in a clinical situation by analysing the PPIX concentration in plasma (a simple 5 min procedure) immediately before light exposure. It is possible that low values could be compensated for by increasing the proposed light dose, and vice versa for high plasma values. Our findings of a minimum necessary plasma concentration do not imply that treatments would be more effective if they are carried out when the plasma level peaks. This might result in an effective tumour necrosis, but would probably damage surrounding normal tissue.

It has been known for some time that tissues incubated *in vitro* with ALA vary in their conversion to PPIX (Sardesai *et al.*, 1964). There is evidence to support the hypothesis that the content of ferrochelatase is low in tumour tissue, possibly being one of the factors responsible for the selectively higher concentration of PPIX (and other porphyrin-related photosensitisers) in tumours compared with most other tissues (Dailey and Smith, 1984). The conversion of human breast tissue explants is about five times more efficient in tumour tissue than in normal tissue (Navone *et al.*, 1988), correlating well with our findings. We also found that conversion efficiency varied in different tissues *in vivo*. Defining safety parameters for correct drug and light dose–a task that, in man, is often very difficult to carry out–can be approximated from animal tissue distribution studies. These data would suggest that some normal tissues (i.e. liver) would be at higher risk of ALA-PDT damage than others.

The extraction procedure we used in this study and our previous study of *m*-THPC (Lofgren *et al.*, 1994), showed higher PPIX levels in muscle than in mucosa. Others have measured PPIX by fluorescence *in vivo* (Pottier *et al.*, 1986), and several studies using fluorescence microscopy have found higher levels in mucosa than muscle (Bedwell *et al.*, 1992; Peng *et al.*, 1992; Loh *et al.*, 1993). We do not have a definitive explanation for this difference, but can speculate on possible reasons. A higher level in epithelial cells might be expected with topical application, where the ALA first contacts the epithelium. With systemic application, either parenteral or oral, the difference is less obvious. However, those studies were done in mice and rats and ours was done in rabbits. It is very possible that the distribution differs with species. In addition, the fluorescence signal would only be detected from the superficial surface of intact tissue, while our extraction process analyses the whole biopsy. Our extraction process is at least 92% efficient (see Materials and methods). The ability to recover such a high level of PPIX from skin confirms that epithelial tissue does not show a preferential reduction of exogenously added PPIX.

It must be recognised that there are major differences in mechanisms of distribution between different photosensitising agents. A photosensitiser such as *m*-THPC comes close to a 'hard drug', which is minimally metabolised before excretion. ALA, in contrast, is a precursor to the active drug, which is synthesised more in some tissues than in others. The active substance is also possibly detoxified largely by a local mechanism by insertion of an iron atom in the molecule, whereas *m*-THPC more likely is excreted in its original form after conjugation to macromolecules by the liver. Therefore, such biodistribution studies must be determined for each drug of interest.

Interspecies dose equivalency is an important factor in translating therapeutic results and toxicity in animals to predicted results in man. Using our measured average rabbit skin size of 0.14 m$^2$, the following calculations can be made. The $K_m$ factor, the ratio of surface area to mass that permits conversions of dosing relationships from one species to another (Spector 1961; Mellet 1969; Van Miert 1986), can be calculated to be 15.7 for rabbits, given a mean measured body weight of 2.2 kg and surface area of 0.14 m$^2$. Multiplying a dose in mg kg$^{-1}$ by the $K_m$ factor gives the corresponding dose in mg m$^{-2}$, e.g. 50 mg kg$^{-1}$ for rabbit will translate to 785 mg m$^{-2}$. To convert from dosages determined in animal studies to a dosage in man, all that is needed is the ratio of the relevant $K_m$ factors. A human adult weighing 60 kg has a $K_m$ factor of 37.5 (Van Miert, 1986). Thus $K_m^{man} \div K_m^{rabbit} = 37.5 \div 15.7 = 2.38$, and a man would require a drug dose $1 \div 2.38$ (42%) as much as the rabbit. For the doses used in this study, equivalent doses in man would be 21 mg kg$^{-1}$, 42 mg kg$^{-1}$ and 84 mg kg$^{-1}$ (13.1 mg m$^{-2}$, 26.3 mg m$^{-2}$ and 53.4 mg m$^{-2}$). Note that this calculation may only be used as a rough guide and not as a recommended dose. The metabolic equivalency in relation to other species, including man, can also be estimated by using the assumption of three-quarter power proportionality of body weight (Van Genderen 1975, Van Os 1982; Van Miert 1986), again only as a rough guide.

The photodegradation experiment carried out in this investigation demonstrated that the concentration of PPIX is significantly reduced (60%) after irradiation with treatment levels of light, i.e. 40–60 J cm$^{-2}$. This reduction in concentration persists for several hours, in contrast to the same situation after injection with *m*-THPC (Ronn and Lofgren, 1994). The difference was also visually apparent because the treated surrounding skin was blanched by treatment with ALA as a photosensitiser, whereas no immediate change in skin colour was usually observed using *m*-THPC under similar irradiation conditions (30–40 J cm$^{-2}$). The cause of the decreased concentration is most likely both a photodegradation or bleaching of the drug itself and a persistent closure of the microcirculatory perfusion of the treated papilloma, preventing reloading of drug and oxygen. We cannot, however, rule out other mechanisms, such as a direct effect on the biosynthesis responsible for the production of PPIX.

In sum, the overall long-term efficiency of papilloma destruction after a single treatment of ALA-PDT is surprisingly high (82%), provided that the above outlined factors are addressed. Taking into consideration the short required interval between administration and treatment and the almost total elimination of active drug within 24 h, we believe that further evaluation of this treatment modality by clinical phase I and II trials is indicated.

**Acknowledgements**

This work was supported by Grant P50 DC 00203 from the National Institute for Deafness and Other Communication Disorders, Bethesda, MD, and grants from the Irving and Helen Schneider family, New York, NY, the Morris S. and Florence H. Bender Foundation, New York, NY, the Otolaryngology Foundation, New York, NY, the Swedish Cancer Foundation, Stockholm, Sweden (2809-B91-02XAB, 03XBB, 01PAC, 02PBC and 03PCC), and Örebro Medical Center Hospital, Örebro, Sweden.

### References

ANDERSON KE. (1990). The prophyrias. In *Hematology*, Williams WJ, Beutler E, Erslev AJ and Lichtman MA. (eds) pp. 722-742. McGraw-Hill: New York.

BEDWELL J, MACROBERT AJ, PHILLIPS D AND BOWN S. (1992). Fluorescence distribution and photodynamic effect of ALA-induced PPIX in the DMH rat colonic tumour model. *Br. J. Cancer*, **65**, 818-824.

BERENBAUM MC, AKANDE SL, BONNETT R, KAUR H, IOANNOU S, WHITE R AND WINFIELD U. (1986). Meso-Tetra(hydroxyphenyl) porphyrins, a new class of potent tumor photosensitizers with favourable selectivity. *Br. J. Cancer*, **54**, 717-725.

BONNETT R, WHITE RD, WINFIELD UJ AND BERENBAUM MC. (1989). Hydroporphyrins of the meso-tetra(hydroxyphenyl) porphyrin series as tumor photosensitizers. *Biochem. J.*, **261**, 277-280.

CAIRNDUFF F, STRINGER MR, HUDSON EJ, ASH DV AND BROWN SB. (1994). Superficial photodynamic therapy with topical 5-aminolaevulinic acid for superficial primary and secondary skin cancer. *Br. J. Cancer*, **69**, 605-608.

DAILEY HA AND SMITH A. (1984). Differential interaction of porphyrins used in photoradiation therapy with ferrochelatase. *Biochem. J.*, **223**, 441-445.

GRANT WE, HOPPER C, MACROBERT AJ, SPEIGHT PM AND BOWN SG. (1993). Photosensitisation with systemic aminolaevulinic acid. *Lancet*, **342**, 147-148.

KENNEDY JC AND POTTIER RH. (1992). Endogenous protoporphyrin IX, a clinically useful photosensitizer for photodynamic therapy. *J. Photochem. Photobiol.*, **14**, 275-292.

KREIDER JW AND BARTLETT GL. (1981). The shope papilloma-carcinoma complex of rabbits: A model system of neoplastic progression and spontaneous regression. *Adv. Cancer Res.*, **35**, 81-110.

LOFGREN LA, RONN AM, ABRAMSON AL, SHIKOWITZ MJ, NOURI M, LEE CJ, BATTI J AND STEINBERG BM. (1994). Photodynamic therapy using meso-tetra(hydroxyphenyl) chlorin: an animal model. *Arch. Otol. Head Neck Surg.*, **120**, 1355-1362.

LOH, MACROBERT AJ, BEDWELL J, REGULA J, KRASNER N AND BOWN SG. (1993). Oral versus intravenous administration of 5-aminolaevulinic acid for photodynamic therapy. *Br. J. Cancer*, **68**, 41-51.

MARCUS SL. (1990). Photodynamic .therapy of human cancer: clinical status, potential and needs. In *Future Directions and Applications in Photodynamic Therapy*, Gomer CJ. (ed.) IS6, pp. 5-56. SPIE Press: Bellingham, Washington.

MELLETT LB. (1969). Comparative drug metabolism. In *Progress in Drug Research*, Vol. 13, Jucker E. (ed.), pp. 136-169. Birkhäuser: Basle.

MULLOOLY VM, ABRAMSON AL AND SHIKOWITZ MJ. (1990). Dihematoporphyrin ether induced photosensitivity in laryngeal papilloma patients. *Lasers Surg. Med.*, **10**, 349-356.

NAVONE NM, FRISARDI AL, RESNIK ER, BATTLE AM AND POLO CF. (1988). Porphyrin biosynthesis in human breast cancer. Preliminary mimetic in vitro studies. *Med. Sci. Res.*, **16**, 61-62.

PENG Q, EVENSEN JF, RIMINGTON C AND MOAN J. (1987). A comparison of different photosensitizing dyes with respect to uptake in C3H tumours and tissues of mice. *Cancer Lett.*, **36**, 1-10.

PENG Q, MOAN J, WARLOE T, NESLAND JM AND RIMINGTON C. (1992). Distribution and photosensitizing efficiency of porphyrins induced by application of exogenous 5-aminolevulinic acid in mice bearing mammary carcinoma. *Int. J. Cancer*, **52**, 433-443.

POTTIER RH, CHOW YFA, LAPLANTE J-P, TRUSCOTT TG, KENNEDY JC AND BIENER LA. (1986). Non-invasive technique for obtaining fluorescence excitation and emission spectra *in vivo*. *Photochem. Photobiol.*, **44**, 679-687.

RIS HB, ALTERMATT HJ, INDERBITZI R, HESS R, NACHBUR B, STEWART JCM, WANG Q, LIM CK, BONNET R, BERENBAUM MC AND ALTHAUS U. (1991). Photodynamic therapy with chlorins for diffuse malignant mesothelioma: initial clinical results. *Br. J. Cancer*, **64**, 1116-1120.

RONN AM AND LOFGREN LA. (1994). meso-Tetra(hydroxyphenyl) chlorin-an in vivo photodegradation study. *Proceedings of Methods for Tumour Treatment and Detection: Mechanisms and Techniques in Photodynamic Therapy*. Part III. *SPIE Proc.*, **2133**, 112-115.

ROUS P AND BEARD JW. (1935). The progression to carcinoma of virus-induced rabbit papillomas (Shope). *J. Exp. Med.*, **62**, 523-548.

SARDESAI VM, WALDMAN J AND ORTEN JM. (1964). A comparative study of porphyrin biosynthesis in different tissues. *Blood*, **24**, 178-186.

SHIMIZU Y, SETSUKO IDA, HIROSHI N AND URATA G. (1978). Excretion of porphyrins in urine and bile after the administration of delta-aminolevulinic acid. *J. Lab. Clin. Med.*, **92**, 795-802.

SPECTOR WS. (1961). *Handbook of Biological Data*. WB Saunders: Philadelphia.

SYVERTON JT. (1952). The pathogenesis of the rabbit papilloma-to-carcinoma sequence. *Ann. NY Acad. Sci.*, **54**, 1441-1446.

TRALAU CJ, BARR H, MACROBERT AJ AND BOWN SG. (1990). Relative merits of porphyrins and pthalocyanine sensitization for photodynamic therapy. In *Photodynamic Therapy of Neoplastic Disease*, Kessel D. (ed.), pp. 263-275. CRC Press: Boca Raton, FL.

VAN MIERT ASJPAM. (1986). Comparatve veterinary pharmacology, toxicology and therapy. In *Proceedings of the 3rd EAVPT Congress*, Ghent 1985, Van Miert ASJPAM and Bogaert MG and Debackere M. (eds) pp. 489-500. MTP Press: Lancaster.

VAN GENDEREN H. (1975). Inleiding over combinaties van geneesmiddelen en over variaties in werking bij verschillende diersoorten. *Tijdschr. Diergeneesk*, **100**, 25-36.

VAN OS JL. (1982). Oestrus control in the bitch with proligestrone. Ph D Thesis, Utrect University.

WOLF P, RIEGER E AND KERL H. (1993). Topical photodynamic therapy with endogenous porphyrins after application of 5-aminolevulinic acid. *J. Am. Acad. Dermatol.*, **28**, 17-21.